# Supplementary figures and images for: Reinvestigating the early embryogenesis in the flatworm Maritigrella crozieri highlights the unique spiral cleavage program found in polyclad flatworms
Source: EvoDevo. 2019 Jun 22;10:12. doi: 10.1186/s13227-019-0126-5 (PMC6588950; doi:10.1186/s13227-019-0126-5)

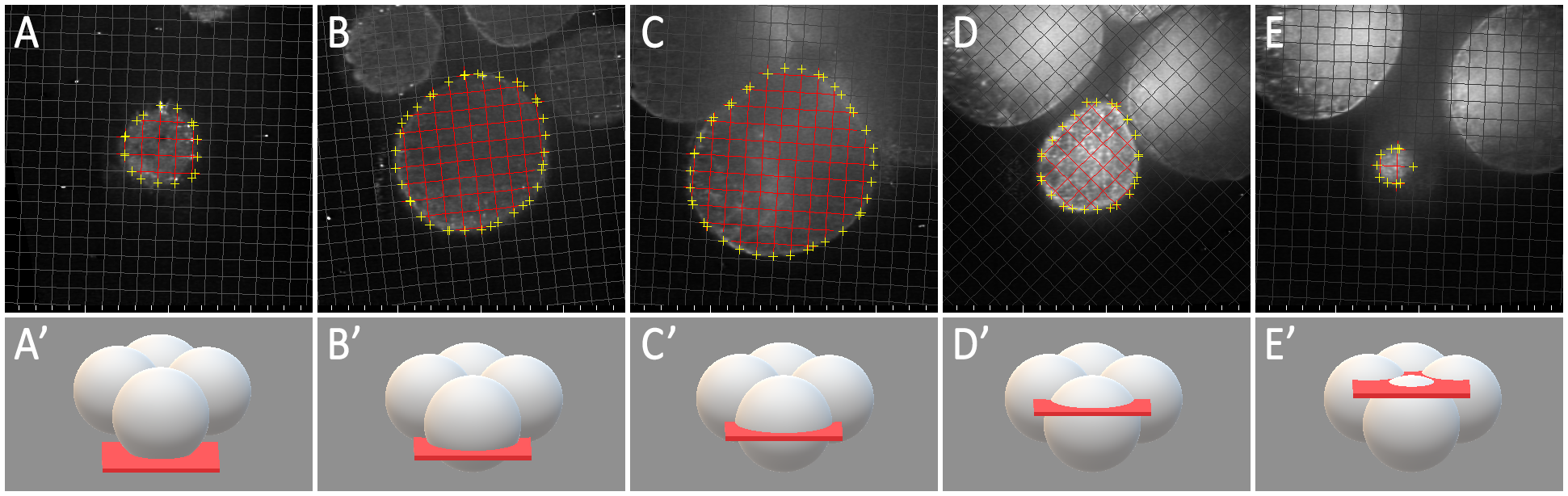

Supplement: Supplementary file 3 — Additional file 3. An example of volume measurements performed on a four-cell stage polyclad flatworm embryo, showing only 5 representative slices within a Z-stack (the original file contains hundreds of slices after image processing is completed). [file 13227_2019_126_MOESM3_ESM.tif]

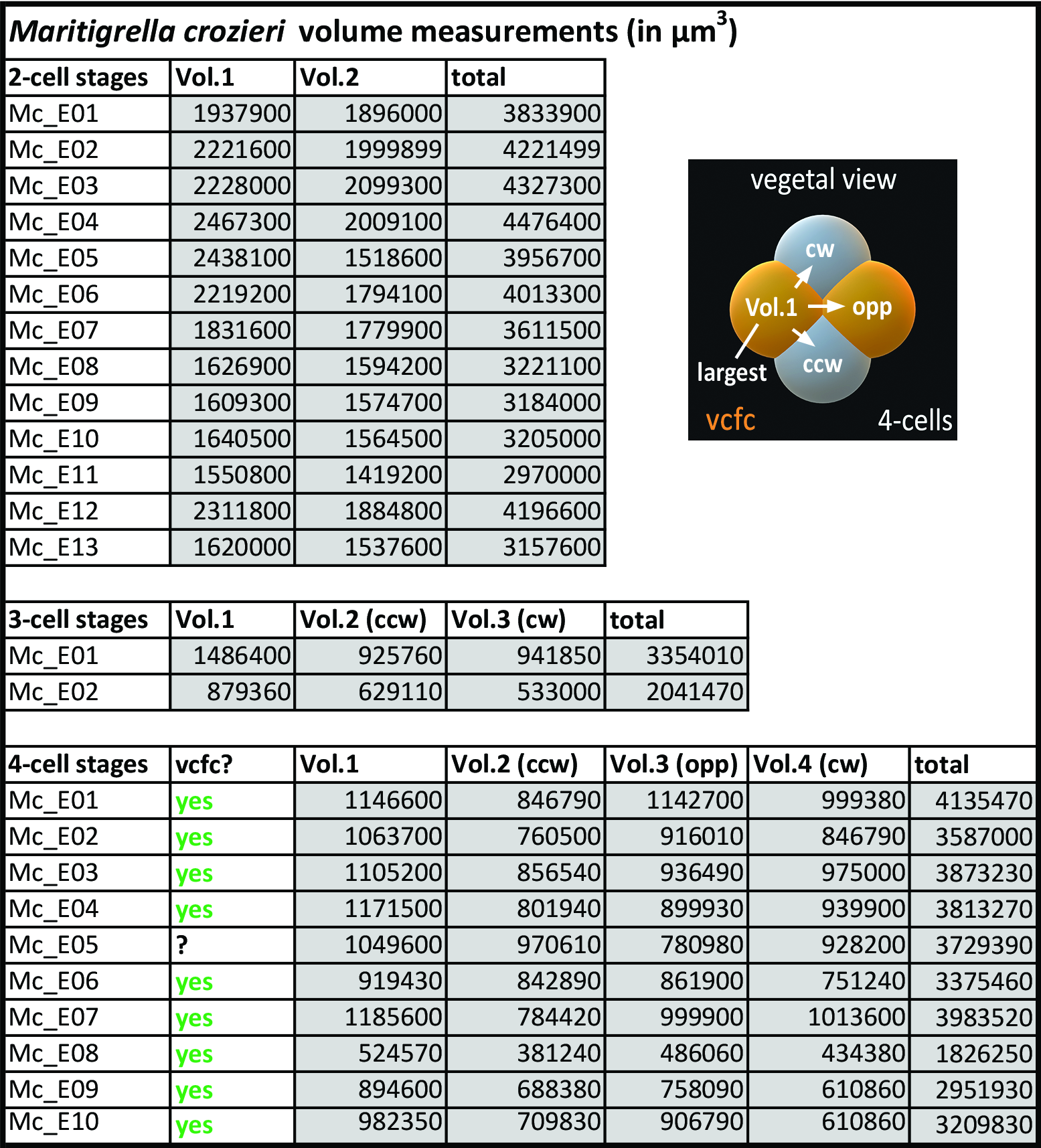

Supplement: Supplementary file 4 — Additional file 4. Table of blastomere volume measurements in 2-, 3- and 4-cell stages. Vol.1 indicates the largest blastomere. In two-cell stages Vol.2 accounts for its sister cell. In four-cell stages Vol.2 corresponds to cells positioned clockwise (cw) of it, Vol. 3 to the cell opposite of it (opp) and Vol.4 counter clockwise (ccw) of it (see schematic embryo inset). The vegetal cross-furrow-cells (vcfc) are shown in orange. [file 13227_2019_126_MOESM4_ESM.tif]

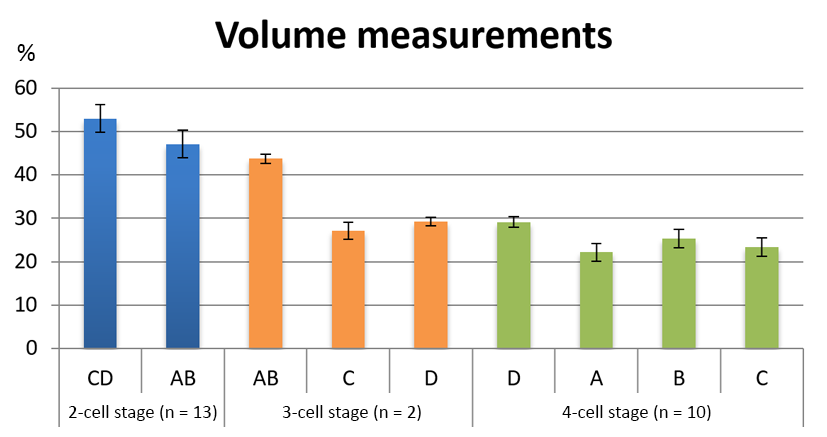

Supplement: Supplementary file 5 — Additional file 5. An average of the volume measurements of 3D reconstructed blastomeres in M. crozieri embryos of the 2-cell, 3-cell and 4-cell stages are shown. The data is based on measurements of individual blastomeres. To provide the data as percentages makes sense as each individual embryo can vary in size. The two-cell stages are indicated as blue columns (n = 13), three-cell stages as orange (n = 2) and four-cell stages as green columns (n = 13). Volumes are given as a percentage of the total volume of the embryo which is 100%. Standard deviations are indicated for smaller blastomeres only. In two-cell stages a 6% difference was noted between the two cells on average. The larger blastomere has been designated as CD. In three-cell stages the two sister blastomeres (C and D) have a larger volume than the remaining sister cell and have been designated as C and D according to a slight volume difference. In four-cell stages the largest blastomere is one of the vegetal cross-furrow cells and has been indicated as D. It is 5.8% larger compared to its sister cell indicated as C. Of the two, remaining sister blastomeres, the size difference is only 3.3% with the larger one indicated as blastomere B. Error bars indicate standard error of the mean. [file 13227_2019_126_MOESM5_ESM.tif]

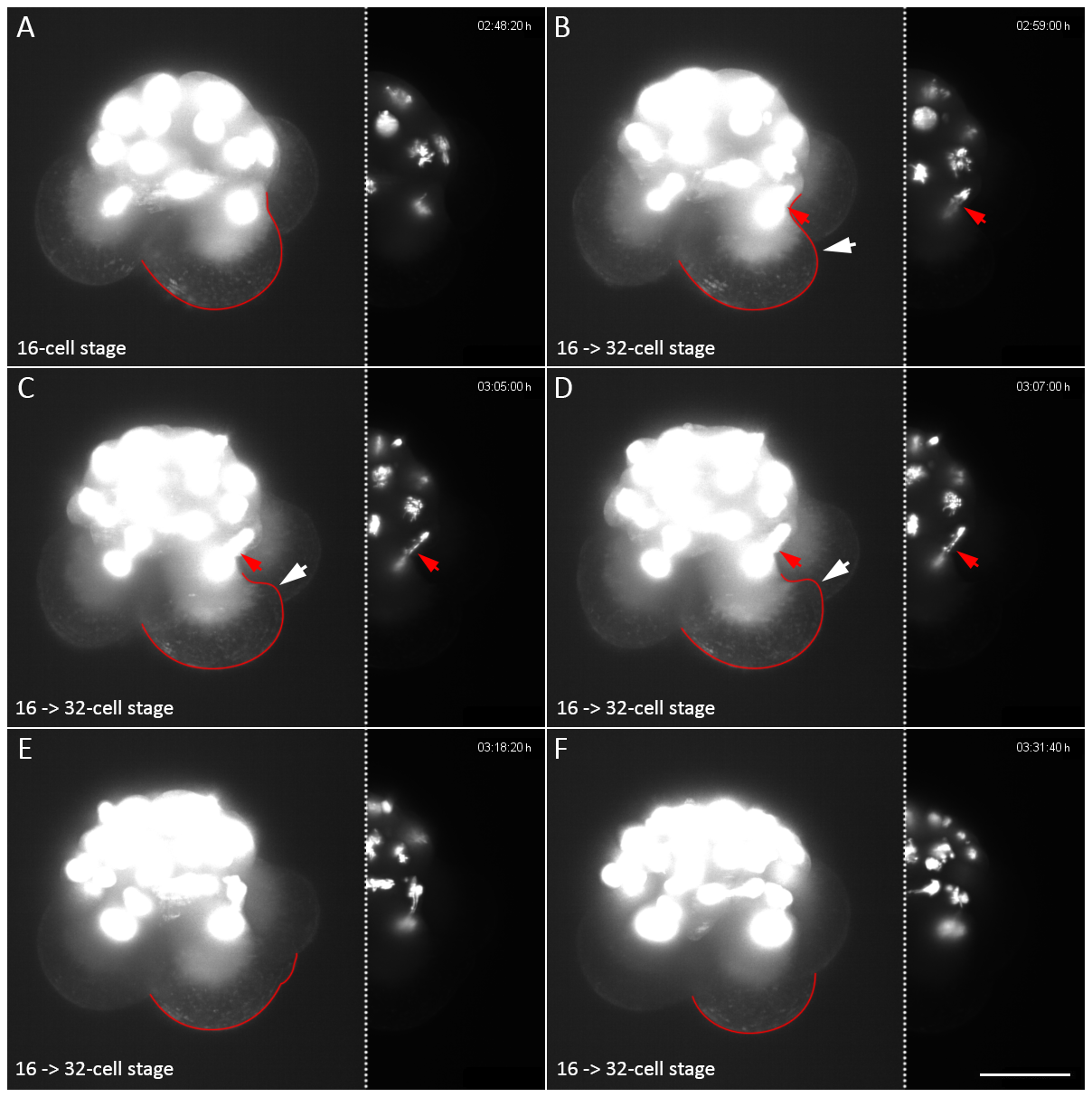

Supplement: Supplementary file 7 — Additional file 7. Cytoplasmic perturbations were imaged with the OpenSPIM in one of the second quartet macromeres during mitosis. On the left the whole embryo is shown with increased brightness to visualize the membranous outlines of the macromeres better. To the right of each embryo, the nuclei of the same embryo are depicted with normal brightness levels. Red arrows point to the same nucleus of the embryo. A red line highlights the outline of the corresponding macromere. The shape deformations caused by the cytoplasmic perturbations of the macromere correlate precisely with the mitotic anaphase and reach a maximum in panel D. Scale bar = 50 µm. [file 13227_2019_126_MOESM7_ESM.tif]

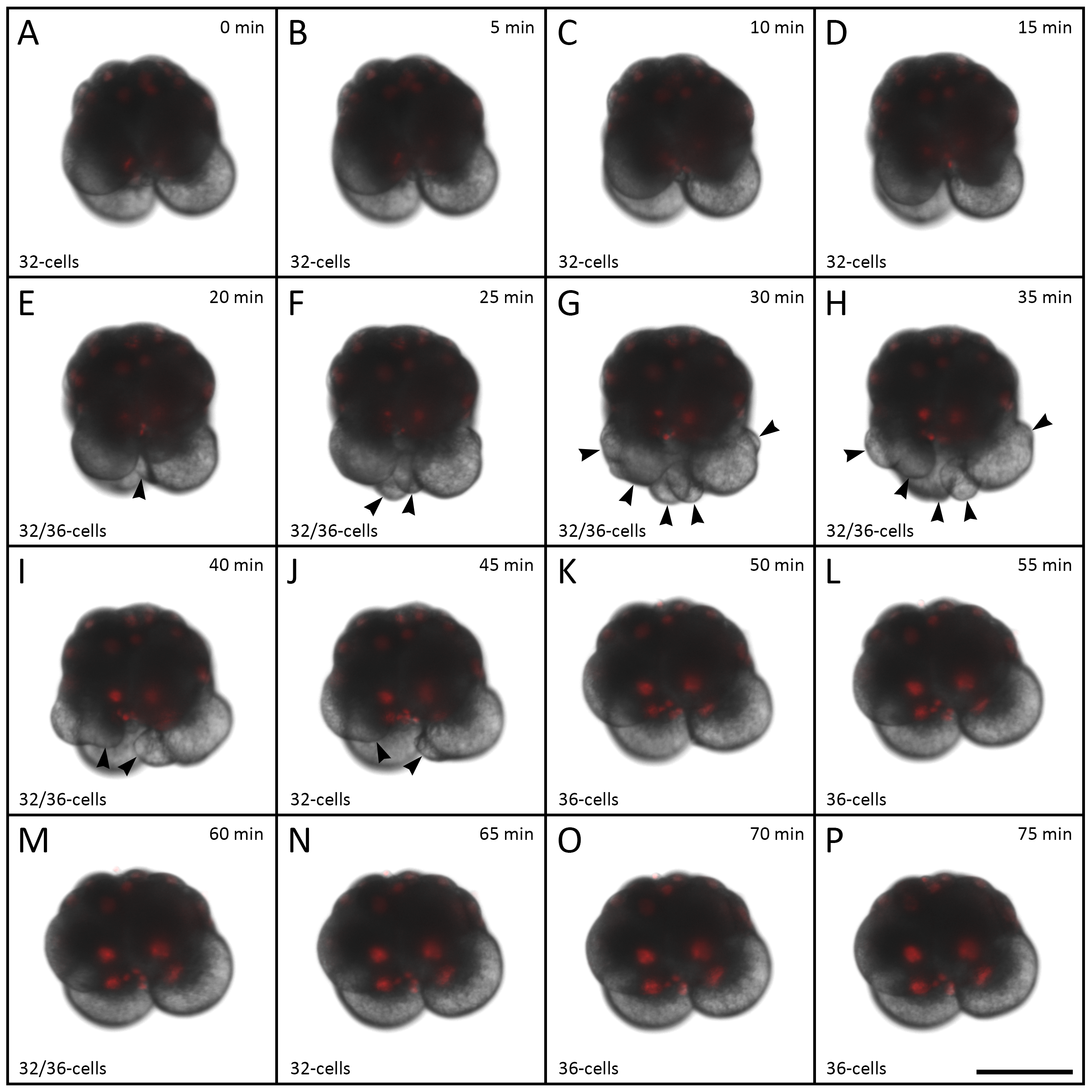

Supplement: Supplementary file 9 — Additional file 9. (A-P) A time-lapse recording showing the formation of the fourth quartet macromeres (4Q) and large micromeres (4q) of a single M. crozieri embryo in 5 min intervals with striking cytoplasmic perturbation activity at the vegetal pole of the embryo (indicated by black arrows). (F-K) 25 min of cytoplasmic perturbations are clearly visible in macromeres 3A–3D. Live imaging was performed under a Zeiss Axio Zoom.V16 Stereo Microscope. Scale bar is 100 µm. [file 13227_2019_126_MOESM9_ESM.tif]
